# Supplementary material for: DNA polymerase α-primase facilitates PARP inhibitor-induced fork acceleration and protects BRCA1-deficient cells against ssDNA gaps
Source: Nat Commun. 2024 Aug 27;15:7375. doi: 10.1038/s41467-024-51667-1 (PMC11350149; doi:10.1038/s41467-024-51667-1)
Supplement: Supplementary file 1 — Supplementary Information [file 41467_2024_51667_MOESM1_ESM.pdf]

Supplementary Figure 1

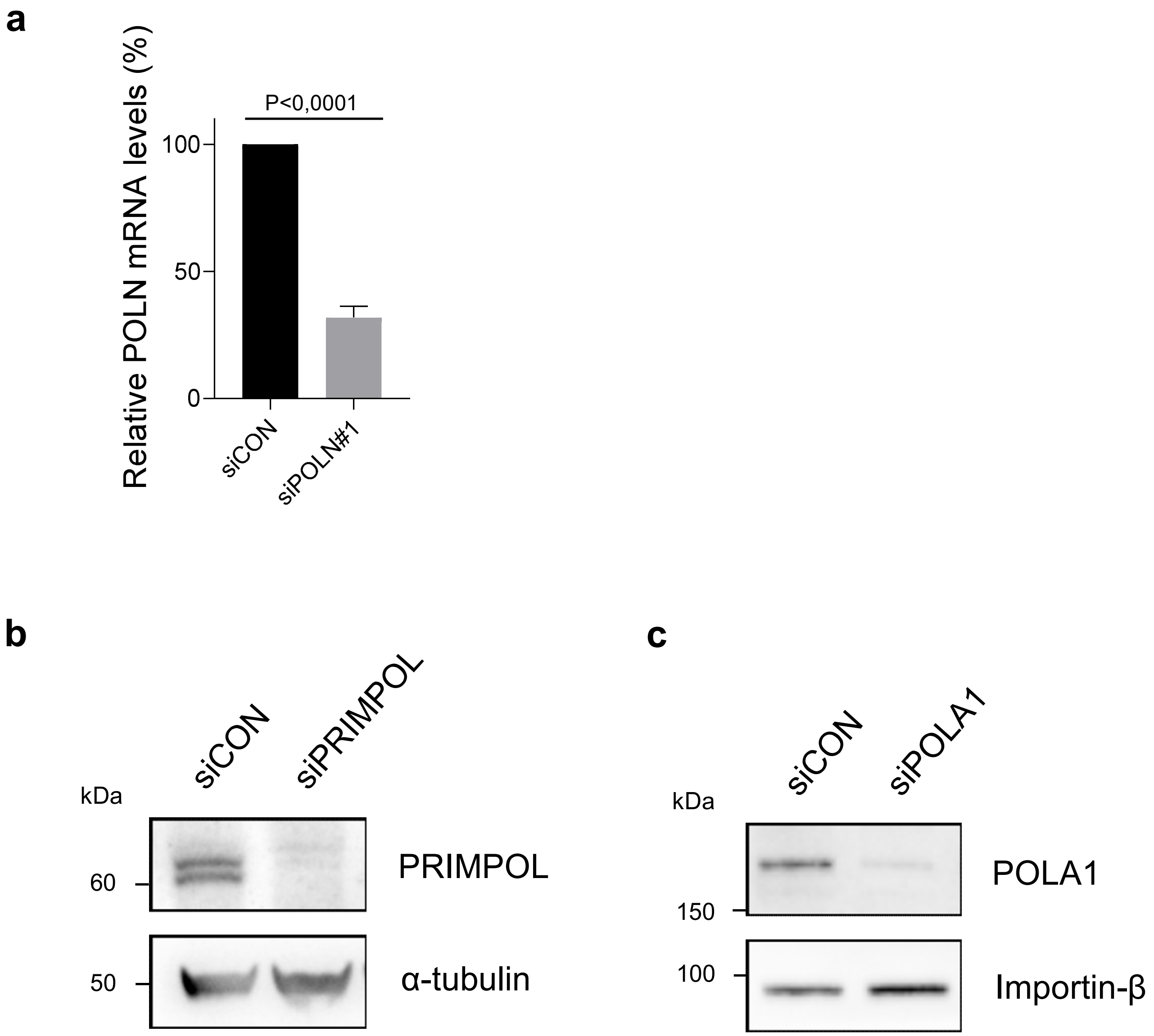

Supplementary Figure 1. POLN, PRIMPOL and POLA1 are efficiently downregulated by siRNAs. a qRT-PCR experiment confirming downregulation of POLN in U2OS cells for the experiment in Figure 1b. The mean values of three independent experiments (n = 3) with standard deviations indicated as error bars are shown. Statistical analysis was conducted by two-tailed Student t- test. **b** Western blot confirming downregulation of PRIMPOL in U2OS cells for the experiment in Figure 1c. **c** Western blot confirming downregulation of POLA1 in U2OS cells for the experiment in Figure 1d. Source data are provided as a Source Data file.

Supplementary Figure 2

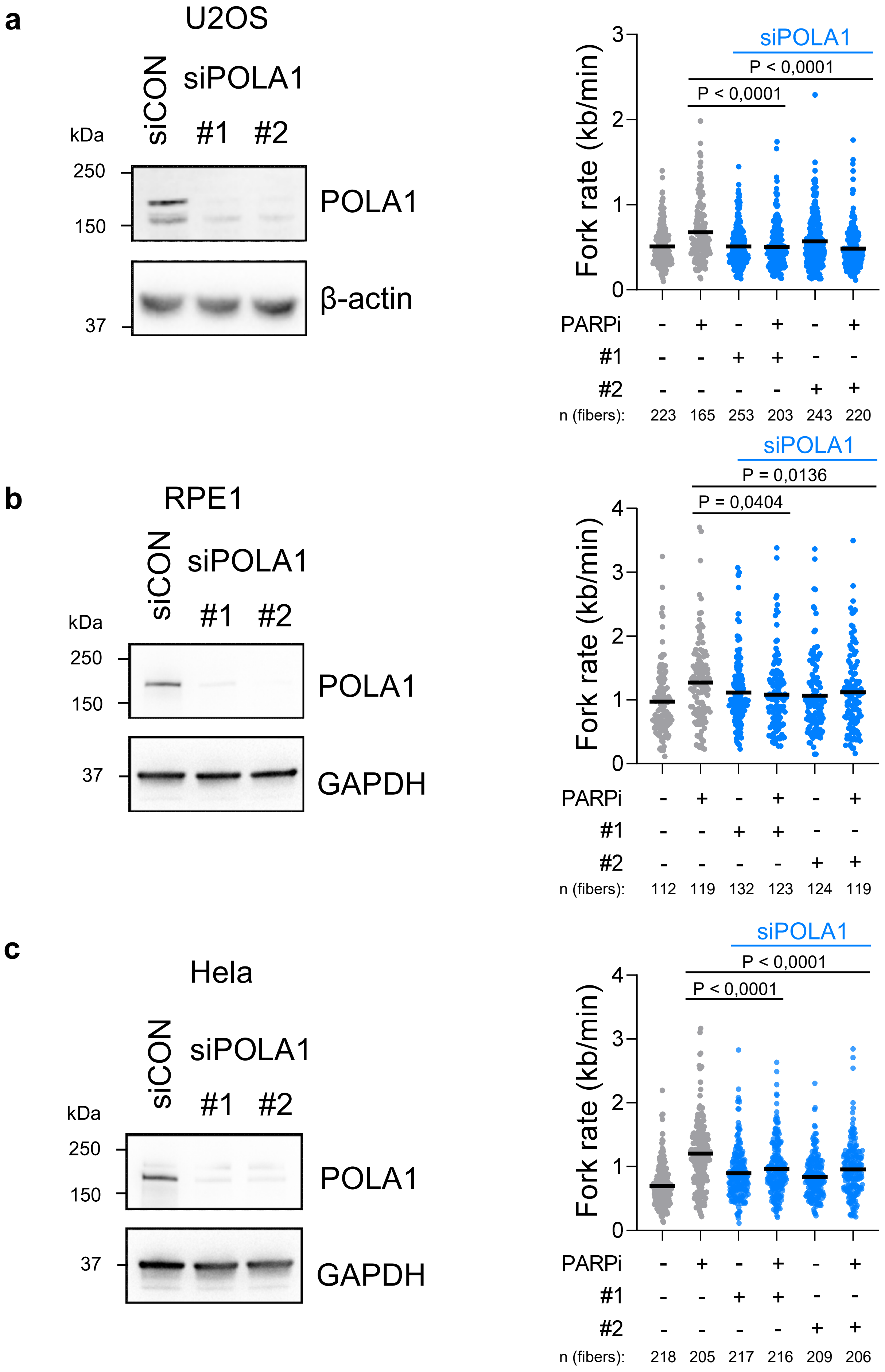

Supplementary Figure 2. POLA1 is required for PARPi-induced fork acceleration. a Western blot (left) showing downregulation of POLA1 in U2OS cells by 2 independent siRNAs and DNA combing assay (right) showing that POLA1 is required for PARPi-induced fork acceleration in U2OS. The scatter plot of fork rates based on IdU tract length is presented, with the mean values marked on the graph. Each dot represents 1 fiber; data are from two independent experiments (n = 2). Statistical analysis was conducted by Kruskal-Wallis test with Dunn's multiple comparisons test. **b** Western blot (left) showing downregulation of POLA1 in RPE1 cells by 2 independent siRNAs and DNA combing assay (right) showing that POLA1 is required for PARPi-induced fork acceleration in RPE1 cells. The scatter plot of fork rates based on IdU tract length is presented, with the mean values marked on the graph. Each dot represents 1 fiber; data are from two independent experiments (n = 2).Statistical analysis was conducted by Kruskal-Wallis test with Dunn's multiple comparisons test. **c** Western blot (left) showing downregulation of POLA1 in Hela cells by 2 independent siRNAs and DNA combing assay (right) showing that POLA1 is required for PARPi-induced fork acceleration in Hela cells. The scatter plot of fork rates based on IdU tract length is presented, with the mean values marked on the graph. Each dot represents 1 fiber; data are from two independent experiments (n = 2). Statistical analysis was conducted by Kruskal-Wallis test with Dunn's multiple comparisons test. Source data are provided as a Source Data file.

Supplementary Figure 3

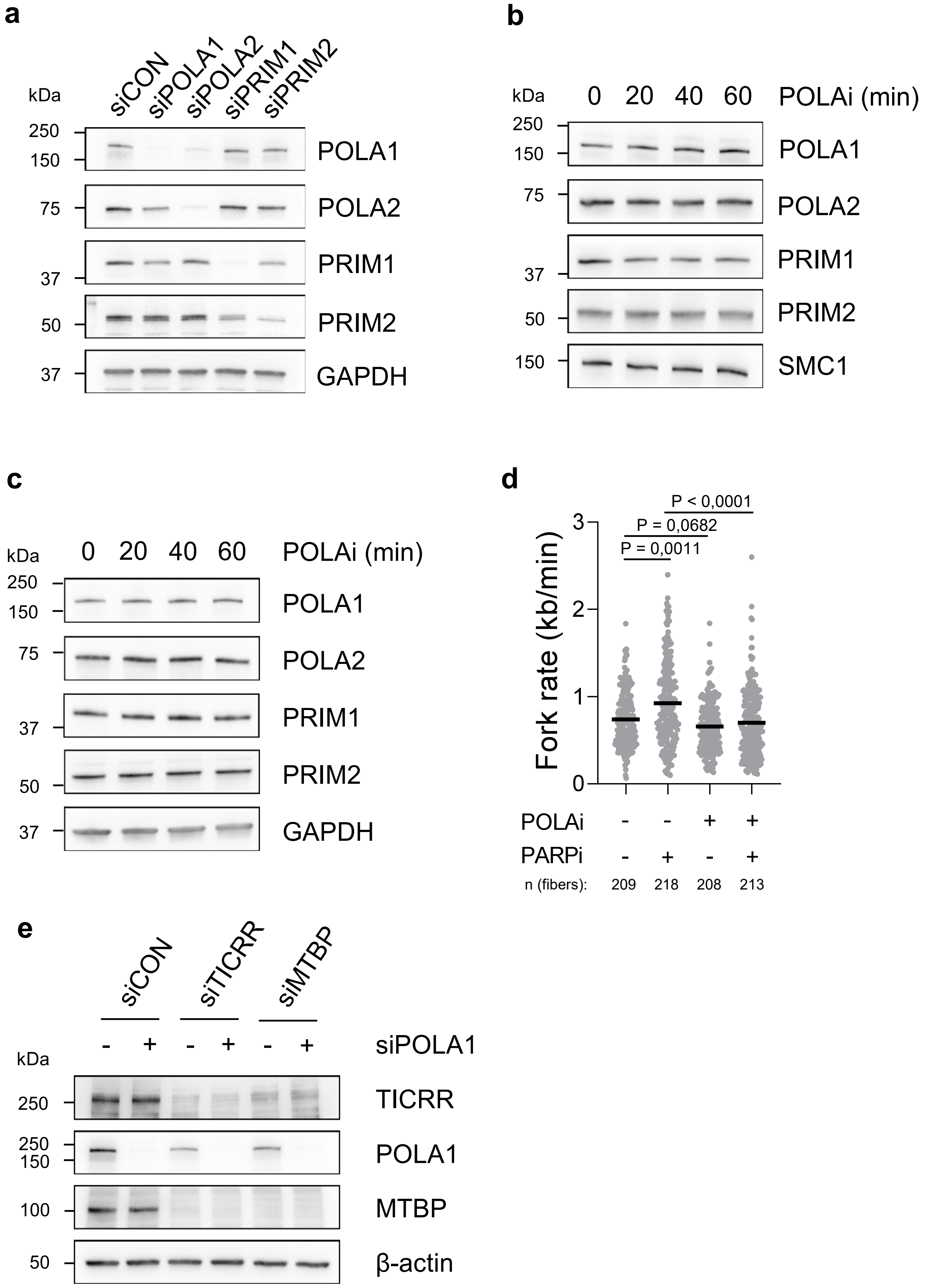

Supplementary Figure 3. POLA1 is specifically required for PARPi-induced fork acceleration. a Western blot confirming knockdown efficiencies of individual POLA subunits in U2OS for an experiment in Figure 3a. **b** Western blot showing that POLA inhibition by 1 μM ST1926 for 20 to 60 minutes in U2OS cells does not affect the stability of POLA complex subunits. **c** Western blot showing that POLA inhibition by 1 μM CD437 for 20 to 60 minutes in U2OS cells does not affect the stability of POLA complex subunits. **d** DNA combing assay showing that inhibition of POLA1 by 2 μM CD437 for 60 minutes prevents PARPi-induced fork acceleration. The scatter plot of fork rates based on IdU tract length is presented, with the mean values marked on the graph. Each dot represents 1 fiber; data are from three independent experiments (n = 3). Statistical analysis was conducted by Kruskal-Wallis test with Dunn's multiple comparisons test. **e** Western blot confirming downregulation of POLA1, TICRR and MTBP in U2OS cells related to the experiment in Figure 1c. Source data are provided as a Source Data file.

Supplementary Figure 4

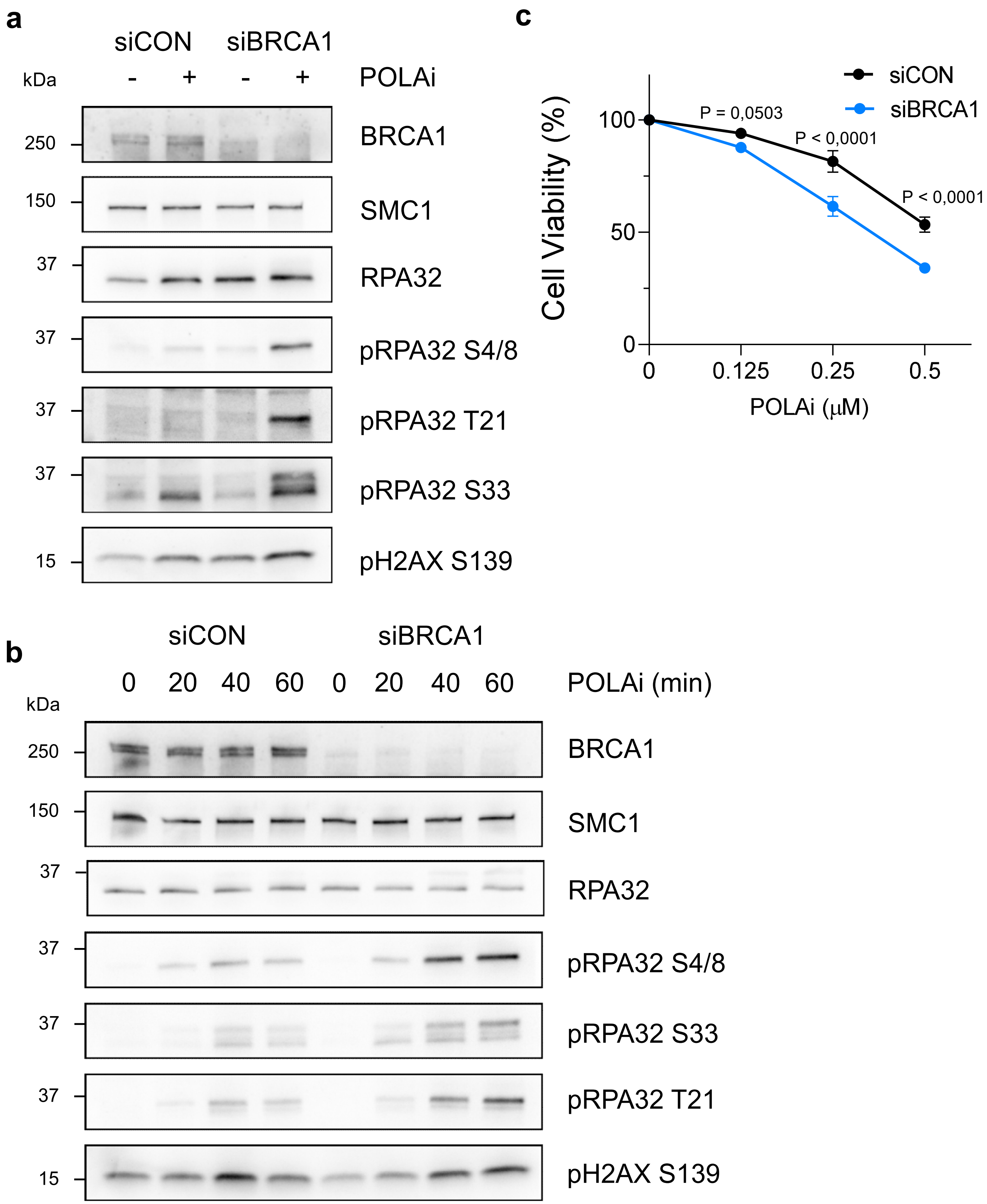

Supplementary Figure 4. BRCA1 downregulation sensitizes cells to POLA1 inhibition. a Western blot showing that POLA1 inhibition (100 nM ST1926 for 16 h) synergistically induces replication stress markers in BRCA1-depleted U2OS cells. **b** Western blot showing that POLA1 inhibition (1 µM ST1926 for 20, 40 or 60 min) induces replication stress markers in BRCA1-depleted U2OS cells. **c** Cellular metabolic activity measured by XTT assay showing that BRCA1 depletion sensitizes U2OS cells to POLA inhibition. The mean values of three independent experiments ($n = 3$) with standard deviations indicated as error bars are shown. Statistical analysis was conducted by one-way ANOVA with Holm-Sidak multiple comparisons test. Source data are provided as a Source Data file.

Supplementary Figure 5

a

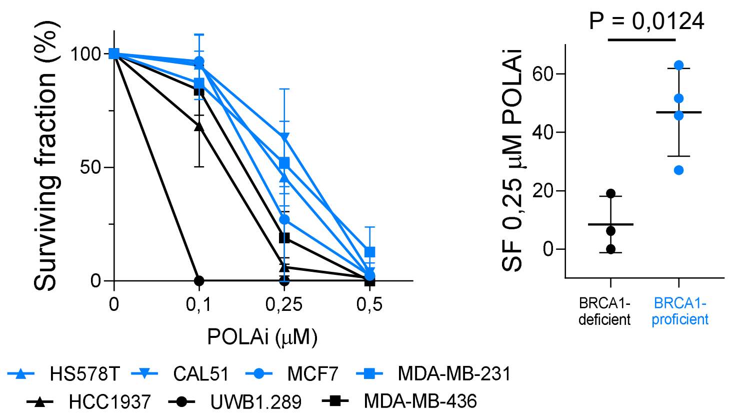

Cell line	SF 0,25 μM (%)	BRCA1
HS578T	45,7	WT
CAL51	63,0	WT
MCF7	27,0	WT
MDA-MB-231	51,6	WT
MDA-MB-436	19,0	defective
HCC1937	6,2	defective
UWB1.289	0	defective

b

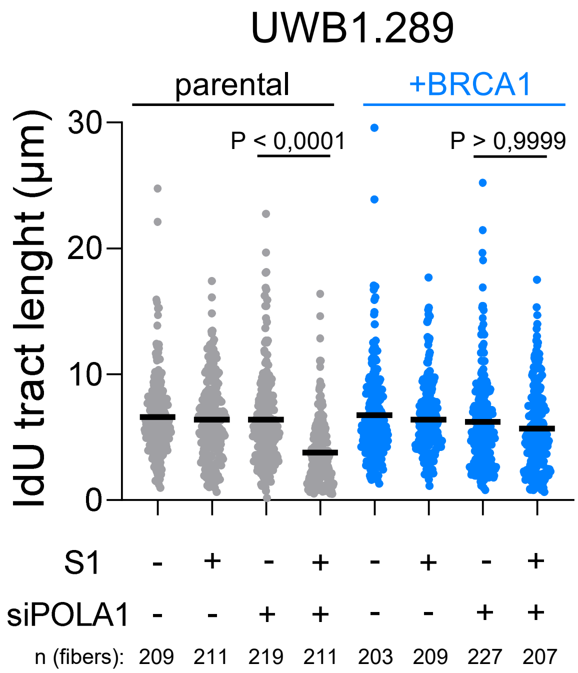

c

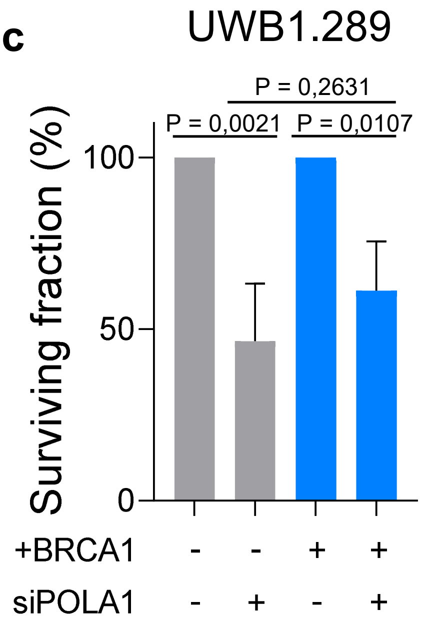

d

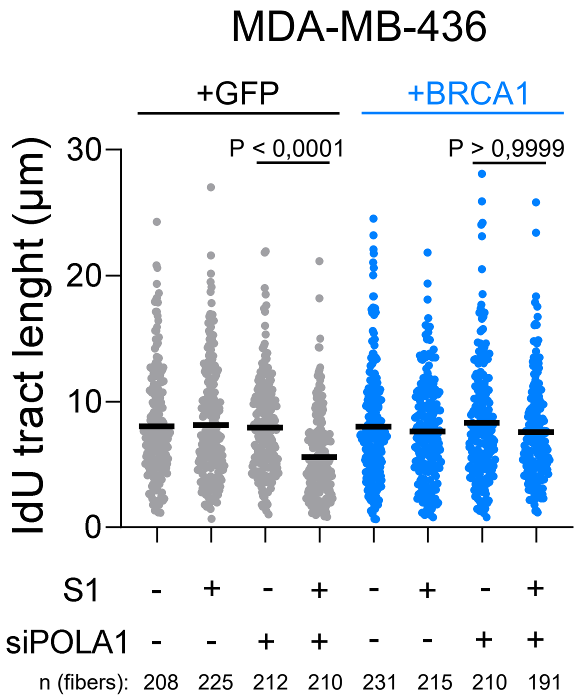

e

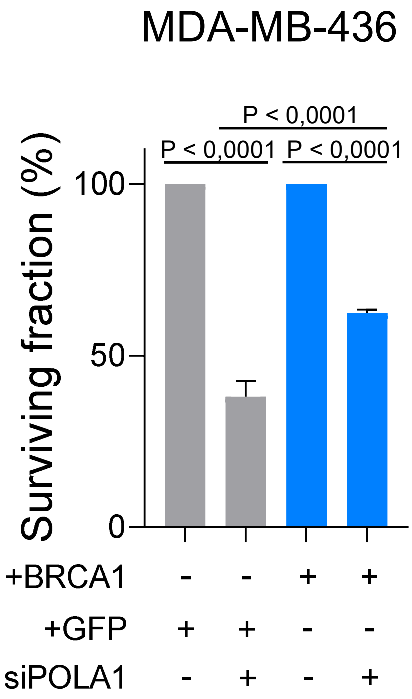

Supplementary Figure 5. BRCA1 deficiency sensitizes cells to POLA1 deregulation. a Clonogenic survival experiment showing that BRCA1-defective cell lines are more sensitive to POLA1 inhibitor ST1926. The surviving fractions (SF) mean values of at least three independent experiments (n = 3) with standard deviations indicated as error bars are shown. Table shows that sensitivity to POLA1 inhibitor ST1926 (0,25 μM) correlates with defects in BRCA1. **b** DNA combing assay with S1 nuclease showing that BRCA1 complementation in UWB1.289 cells prevents formation of ssDNA gap accumulation induced by POLA1 knockdown. Scatter plot of fork IdU tract lengths is presented, with the mean values marked on the graph. Each dot represents 1 fiber; data are from two independent experiments (n = 2). Statistical analysis was conducted by Kruskal-Wallis test with Dunn's multiple comparisons test. **c** Clonogenic survival experiment showing that BRCA1 complementation makes UWB1.289 cells resistant to POLA1 downregulation. The mean values of three independent experiments (n = 3) with standard deviations indicated as error bars are shown. Statistical analysis was conducted by one-way ANOVA with Holm-Sidak multiple comparisons test. **d** DNA combing assay with S1 nuclease showing that BRCA1 complementation in MDA-MB-436 cells prevents formation of ssDNA gap accumulation induced by POLA1 knockdown. Scatter plot of fork IdU tract lengths is presented, with the mean values marked on the graph. Each dot represents 1 fiber; data are from two independent experiments (n = 2). Statistical analysis was conducted by Kruskal-Wallis test with Dunn's multiple comparisons test. **e** Clonogenic survival experiment showing that BRCA1 complementation makes MDA-MB-436 cells resistant to POLA1 downregulation. The mean values of three independent experiments (n = 3) with standard deviations indicated as error bars are shown. Statistical analysis was conducted by one-way ANOVA with Holm-Sidak multiple comparisons test. Source data are provided as a Source Data file.
