# Supplementary material for: DNA polymerase α-primase facilitates PARP inhibitor-induced fork acceleration and protects BRCA1-deficient cells against ssDNA gaps
Source: Nat Commun. 2024 Aug 27;15:7375. doi: 10.1038/s41467-024-51667-1 (PMC11350149; doi:10.1038/s41467-024-51667-1)
Supplement: Supplementary file 3 — Description of Additional Supplementary Files [file 41467_2024_51667_MOESM3_ESM.pdf]

Description of Additional Supplementary Files

File Name: Supplementary Data 1

Description: This file includes information about all antibodies used in the study.

IB - immunoblotting

IF - immunofluorescence

DC - DNA combing
