# Supplementary material for: DNA polymerase α-primase facilitates PARP inhibitor-induced fork acceleration and protects BRCA1-deficient cells against ssDNA gaps
Source: Nat Commun. 2024 Aug 27;15:7375. doi: 10.1038/s41467-024-51667-1 (PMC11350149; doi:10.1038/s41467-024-51667-1)
Supplement: Supplementary file 4 — Supplementary Data 1 [file 41467_2024_51667_MOESM4_ESM.docx]

*Antibody Company Catalogue nr. Dilution and Application*

PRIMPOL Proteintech 29824-1-AP 2000x for IB

PRIMPOL Novus NBP2-67217 250x for IB

POLA1 Sigma-Aldrich HPA002947 1000x for IB

POLA2 Invitrogen PA5-58015 2000x for IB

PRIM1 Cell Signaling #4725S 1000x for IB

PRIM2 Invitrogen PA5-88189 1000x for IB

β-Actin Santa Cruz Bt. sc-47778 500x for IB

α-tubulin Santa Cruz Bt. sc-8035 1000x for IB

Importin β Abcam ab2811 5000x for IB

GAPDH Cell Signaling #2118S 1500x for IB

BrdU Abcam ab6326 50x for DC, 100x for IF

BrdU BD Biosciences BD347580 10x for DC

TICRR Rockland 600-401-FE3 500x for IB

MTBP Santa Cruz sc-137201 1000x for IB

RPA32 Abcam ab2175 1000x for IB, 500x for IF

γH2AX Cell Signaling #9718 500x for IB, 500x for IF

phosRPA32 S4/8 Bethyl A300-245A 4000x for IB

phosRPA32 T21 Abcam ab61065 250x for IB

phosRPA32 S33 Bethyl A300-246A 1500x for IB

BRCA1 Santa Cruz Bt. sc-6954 200x for IB

SMC1 Abcam ab9262 5000x for IB
