# Supplementary material for: DNA polymerase α-primase facilitates PARP inhibitor-induced fork acceleration and protects BRCA1-deficient cells against ssDNA gaps
Source: Nat Commun. 2024 Aug 27;15:7375. doi: 10.1038/s41467-024-51667-1 (PMC11350149; doi:10.1038/s41467-024-51667-1)
Supplement: Supplementary file 5 — Reporting Summary [file 41467_2024_51667_MOESM5_ESM.pdf]

nature portfolio

Corresponding author(s): Pavel Moudry

Last updated by author(s): Jul 26, 2024

Reporting Summary

Nature Portfolio wishes to improve the reproducibility of the work that we publish. This form provides structure for consistency and transparency in reporting. For further information on Nature Portfolio policies, see our Editorial Policies and the Editorial Policy Checklist.

Statistics

For all statistical analyses, confirm that the following items are present in the figure legend, table legend, main text, or Methods section.

n/a	Confirmed	
☐	☒	The exact sample size (n) for each experimental group/condition, given as a discrete number and unit of measurement
☐	☒	A statement on whether measurements were taken from distinct samples or whether the same sample was measured repeatedly
☐	☒	The statistical test(s) used AND whether they are one- or two-sided<br>*Only common tests should be described solely by name; describe more complex techniques in the Methods section.*
☒	☐	A description of all covariates tested
☒	☐	A description of any assumptions or corrections, such as tests of normality and adjustment for multiple comparisons
☐	☒	A full description of the statistical parameters including central tendency (e.g. means) or other basic estimates (e.g. regression coefficient) AND variation (e.g. standard deviation) or associated estimates of uncertainty (e.g. confidence intervals)
☐	☒	For null hypothesis testing, the test statistic (e.g. F, t, r) with confidence intervals, effect sizes, degrees of freedom and P value noted<br>*Give P values as exact values whenever suitable.*
☒	☐	For Bayesian analysis, information on the choice of priors and Markov chain Monte Carlo settings
☒	☐	For hierarchical and complex designs, identification of the appropriate level for tests and full reporting of outcomes
☒	☐	Estimates of effect sizes (e.g. Cohen's d, Pearson's r), indicating how they were calculated

Our web collection on statistics for biologists contains articles on many of the points above.

Software and code

Policy information about availability of computer code

Data collection	1) DNA combing: Zen 2 blue edition (Zeiss)<br>2) Immunofluorescence: scanR Acquisition 3.3.0 (Olympus)<br>3) Immunoblotting: Image Lab 6.1.0 (Bio-Rad)
Data analysis	1) DNA combing: ImageJ 1.53k, Excel v2310 (Microsoft)<br>2) Immunofluorescence: scanR Analysis 3.4.0 (Olympus)<br>3) Statistics and Data Figure Panel: GraphPad Prism 9.3.1 (Dotmatics), Excell v2310 (Microsoft)<br>4) Inkscape 1.3.2.

For manuscripts utilizing custom algorithms or software that are central to the research but not yet described in published literature, software must be made available to editors and reviewers. We strongly encourage code deposition in a community repository (e.g. GitHub). See the Nature Portfolio guidelines for submitting code & software for further information.

Data

Policy information about availability of data

All manuscripts must include a data availability statement. This statement should provide the following information, where applicable:

- Accession codes, unique identifiers, or web links for publicly available datasets
- A description of any restrictions on data availability
- For clinical datasets or third party data, please ensure that the statement adheres to our policy

The data generated during this study are available within the paper and its Supplementary Information and Source Data files.

Research involving human participants, their data, or biological material

Policy information about studies with human participants or human data. See also policy information about sex, gender (identity/presentation), and sexual orientation and race, ethnicity and racism.

Reporting on sex and gender	Not applicable
Reporting on race, ethnicity, or other socially relevant groupings	Not applicable
Population characteristics	Not applicable
Recruitment	Not applicable
Ethics oversight	Not applicable

Note that full information on the approval of the study protocol must also be provided in the manuscript.

Field-specific reporting

Please select the one below that is the best fit for your research. If you are not sure, read the appropriate sections before making your selection.

☒ Life sciences ☐ Behavioural & social sciences ☐ Ecological, evolutionary & environmental sciences

For a reference copy of the document with all sections, see nature.com/documents/nr-reporting-summary-flat.pdf

Life sciences study design

All studies must disclose on these points even when the disclosure is negative.

Sample size	Due to the nature of the method presented in the manuscript no statistical method was necessary to determine the sample size. Sample size was determined based on prevous experiments and relevant literature in the field (e.g. Maya-Mendoza et al., PMID: 29950726; Cong et al., PMID: 34216544; Hale et al., PMID: 37805499; Leung et al., PMID: 37454295). Statistical significance was obtained with this sample size.
Data exclusions	No data were excluded from the analysis.
Replication	All experiments were replicated at least twice to reliably support conclusions stated in the manuscript. All results were reproducible.
Randomization	We tested low number of different treatment condition in cells of the same age. All samples were analyzed by the same standard protocol. Randomization was not relevant for this type of study. Samples were allocated to control group or genetic treatment group.
Blinding	For DNA combing and Immunofluorescence assays, samples were tested at least once under blinding conditions, and results were reproduced.

Reporting for specific materials, systems and methods

We require information from authors about some types of materials, experimental systems and methods used in many studies. Here, indicate whether each material, system or method listed is relevant to your study. If you are not sure if a list item applies to your research, read the appropriate section before selecting a response.

Materials & experimental systems

n/a	Involved in the study
☐	☒ Antibodies
☐	☒ Eukaryotic cell lines
☒	☐ Palaeontology and archaeology
☒	☐ Animals and other organisms
☒	☐ Clinical data
☒	☐ Dual use research of concern
☒	☐ Plants

Methods

n/a	Involved in the study
☒	☐ ChIP-seq
☒	☐ Flow cytometry
☒	☐ MRI-based neuroimaging

Antibodies

Antibodies used

PRIMPOL (Proteintech, 29824-1-AP, polyclonal, lot: 00107145, IB: 2000x)
PRIMPOL (Novus, 2-67217, clone: 10-B3, lot: HM1008, IB: 250x)
POLA1 (Sigma-Aldrich, HPA002947, polyclonal, lot: A95954 IB: 1000x)
POLA2 (Invitrogen, PA5-58015, polyclonal, lot: R35538, IB: 2000x)
PRIM1 (Cell Signaling, #4725S, clone: 8G10, lot: 1, IB: 1000x)
PRIM2 (Invitrogen, PA5-88189, polyclonal, lot: XB3511498A, IB: 1000x)
β-Actin (Santa Cruz Biotechnology, sc-47778, clone: C4, lot: C1620, IB: 500x)
α-tubulin (Santa Cruz Biotechnology, sc-8035, clone: TU-02, lot: D1317, IB: 1000x)
Importin β (Abcam, ab2811, clone: 3E9, lot: GR217711-3, IB:5000x)
GAPDH (Cell Signaling, #2118S, clone: 14C10, lot: 14, IB: 1500x)
BrdU (Abcam, ab6326, clone: BU1/75 (ICR1), lot: 1009715-6, IF: 100, DNA combing: 50x)
BrdU (BD Biosciences, BD347580, clone: B44, lot: 2192362, DNA combing: 10x)
TICRR (Rockland, 600-401-FE3, polyclonal, lot: 43163, IB: 500x)
MTBP (Santa Cruz, sc-137201, clone: B-5, lot: E1418, IB: 1000x)
RPA32 (Abcam, ab2175, clone: 9H8, lot: GR3453257-1, IF: 500x, IB: 1000x)
γH2AX (Cell Signaling, #9718, clone: 20E3, lot: 21, IF: 500x, IB: 500x)
phosphoRPA32 S4/8 (Bethyl, A300-245A, polyclonal, lot: 4, IB: 4000x)
phosphoRPA32 T21 (Abcam, ab61065, polyclonal, lot: GR54003-4, IB: 250x)
phosphoRPA32 S33 (Bethyl, A300-246A, polyclonal, lot: 7, IB: 1500x)
BRCA1 (Santa Cruz Biotechnology, sc-6954, clone: D-9, lot: A1018, IB: 250x)
SMC1 (Abcam, ab9262, polyclonal, lot: 1004100-2, IB: 5000x)

Validation

PRIMPOL, POLA1, POLA2, PRIM1, PRIM2, TICRR, MTBP and BRCA1 antibodies were validated by immunoblitting using siRNA-mediated knockdown as negative control. The data are presented in the manuscript (Supplementary Fig. 1bc, Supplementary Fig. 2abc, Supplementary Fig. 3ae, Supplementary Fig. 4ab).

β-Actin (Santa Cruz Biotechnology, sc-47778) antibody used for immunobloting was validated by the company, cited in 13785 publications and our work (Turi et al., PMID: 29143558, Moudry et al., PMID: 34611297, Lukac et al., PMID: 36260751).

α-tubulin (Santa Cruz Biotechnology, sc-8035) antibody used for immunobloting was validated by the company, cited in 1445 publications and our work (Moudry et al., PMID: 26811421 and Moudry et al., PMID: 34611297).

Importin β (Abcam, ab2811) antibody used for immunobloting was validated by the company, cited in 52 publications and our work (Moudry et al., PMID: 22075984 and Moudry et al., PMID: 26811421).

GAPDH (Cell Signaling, #2118S) antibody antibody used for immunobloting was walidated by the company and cited in 7856 publications.

SMC1 (Abcam, ab9262) antibody used for immunobloting was walidated by the company and cited in 36 publications.

BrdU (Abcam, ab6326 and BD Biosciences, BD347580) antibodies used for DNA combing were validated by producers and by the field. We used those antibodies in our previousl publications (e.g. Maya-Mendoza et al., PMID: 29950726; Moudry et al., PMID: 34611297; Lukac et al., PMID: 36260751).

γH2AX (Cell Signaling, #9718) antibody used for immunofluorescence was validated by the company and cited in 2136 publications.
RPA32 (Abcam, ab2175) antibody used for immunofluorescence was validated by the company and cited in 182 publications including our work (Moudry et al., PMID: 26811421; Maya-Mendoza et al., PMID: 29950726).
BrdU (Abcam, ab6326) antibody used for immunofluorescence was validated by the company and cited in 1397 publications.

Eukaryotic cell lines

Policy information about cell lines and Sex and Gender in Research

Cell line source(s)

U2OS (ATCC Number: HTB-96), Hela (ATCC Number: CCL-2), RPE1 (ATCC Number: CRL-4000), UWB1.289 (ATCC Number: CRL-2945) and UWB1.289+BRCA1 (ATCC Number: CRL-2946) cell lines were obtained from ATCC. MDA-MB-436 cells reconstituted with GFP or BRCA1 were recieved from Neil Johnson (Fox Chase Cancer Center).

Authentication	All cell lines were authenticated by STR method.
Mycoplasma contamination	All cell lines were tested for mycoplasma contamination and the tests were negative.
Commonly misidentified lines (See ICLAC register)	None of the cell lines used are listed on the ICLAC register.

Plants

Seed stocks	*Report on the source of all seed stocks or other plant material used. If applicable, state the seed stock centre and catalogue number. If plant specimens were collected from the field, describe the collection location, date and sampling procedures.*
Novel plant genotypes	*Describe the methods by which all novel plant genotypes were produced. This includes those generated by transgenic approaches, gene editing, chemical/radiation-based mutagenesis and hybridization. For transgenic lines, describe the transformation method, the number of independent lines analyzed and the generation upon which experiments were performed. For gene-edited lines, describe the editor used, the endogenous sequence targeted for editing, the targeting guide RNA sequence (if applicable) and how the editor was applied.*
Authentication	*Describe any authentication procedures for each seed stock used or novel genotype generated. Describe any experiments used to assess the effect of a mutation and, where applicable, how potential secondary effects (e.g. second site T-DNA insertions, mosiacism, off-target gene editing) were examined.*
